# Multiform-based Baduanjin exercise prevention and treatment for idiopathic pulmonary fibrosis: study protocol for a randomized controlled trial

**DOI:** 10.1186/s12906-023-03974-1

**Published:** 2023-05-12

**Authors:** Zenan Wu, Zehao Hu, Shiwen Ke, Lisha Mo, Mingliang Qiu, Guoshuang Zhu, Wei Zhu, Liangji Liu

**Affiliations:** 1 The Clinical Medical School Jiangxi University of Traditional Chinese Medicine, Nanchang, China; 2 The Affiliated Hospital of Jiangxi University of Traditional Chinese Medicine, Nanchang, China; 3 The Second Clinical Medical School, Guangzhou University of Chinese Medicine, Guangzhou, Guangdong China

**Keywords:** Idiopathic pulmonary fibrosis, Baduanjin, Lung function, RCT

## Abstract

**Introduction:**

In this study, we will combine the traditional Baduanjin with Yijin Jing and Wuqinxi to create an optimized Baduanjin exercise program with three different forms (vertical, sitting, and horizontal) to adapt to idiopathic pulmonary fibrosis (IPF) patients in vairous stages of the disease. The purpose of this study is to explore and compare the therapeutic effects of this multi-form Baduanjin, traditional Baduanjin, and resistance training on lung function and limb motor function in IPF patients. The goal of this study is to prove a novel optimal exercise prescription strategy of Baduanjin exercise for improving and protecting lung function in IPF patients.

**Methods/design:**

A single-blind and randomized controlled trial is used to conduct this study, while the randomization list will be generated using a computerized random number generator and opaque sealed envelopes with group allocation will be prepared. It will be strictly followed to blind the outcome assessors. and until the experiment’s conclusion, participants won’t know which group they are enrolled in. Patients between the ages of 35 and 80 who have stable diseases and have not regularly practiced Baduanjin exercise in the past will be included. They are divvied up into the following five groups at random: (1) The conventional care group (control group, CG), (2) The traditional Baduanjin exercise group (TG), (3) The modified Baduanjin exercise group (IG), (4) The resistance exercise group (RG) (5) The modified Baduanjin exercise combined with resistance exercise group (IRG). Those CG participants only received the usual treatment, while TC, IG, and RG participants exercised 1 h twice a day for 3 months. MRG participants will have a 3-month intervention with 1 h of Modified Baduanjin Exercise and 1 H of Resistance Training for each day. Every week, all groups underwent will supervis one-day training, with the exception of the control group. The Pulmonary Function Testing (PFT), HRCT, and 6MWT are the main outcome variables. The St. George Respiratory Questionnaire and mMRC are used as secondary outcome measures.

**Discussion:**

This study may produce a new Baduanjin exercise prescription that is user-friendly, simple to execute, more targeted, and adaptable. Because it consists of three forms, including vertical, sitting, and horizontal, it is more adaptable to the various disease stages and actual situations of IPF patients and may compensate for the shortcomings of conventional pulmonary rehabilitation and traditional Baduanjin.

**Trial registration:**

Chinese Clinical Trial Registry, ChiCTR2200055559. Registered on 12 January 2022.

## Background

Idiopathic pulmonary fibrosis (IPF) is a worldwide respiratory disease, which seriously affects the quality of life and workability of patients. It is characterized by dyspnea and progressive deterioration of pulmonary function with a poor prognosis. The incidence of IPF increases with age, with the typical onset of dyspnea between 60 and 70 years of age [1, 2]. The pathogenesis of IPF is still unclear, while the lesion site is limited to the lungs. All adults with unexplained chronic labor dyspnea, cough, double basal artery aspirational popping sound, and/or clubbing fingers should be evaluated for IPF [3]. Currently, the incidence of idiopathic pulmonary fibrosis is increasing globally, with an average incidence of 3 cases per 100,000 people per country. According to current data, rates of stomach, liver, testicular, and cervical cancers are comparable [4]. Currently, Pirfenidone or Nintedanib monotherapy is the most effective treatment for IPF. These medications carry certain risks, have limited utility, and the treatment process is fraught with uncertainty. For instance, more than 20% of patients have discontinued Nintedanib due to adverse events such as diarrhea and liver dysfunction. [5]. Innovative therapies and drug combinations hold great promise for the future, and the effects of non-drug interventions, including various physical therapies, have been assessed and reported [6]. Studies have shown that aerobic and respiratory training appears to complement exercise training and improve dyspnea and health-related quality of life in IPF patients [7]. In Laboratory Animal Research, it is suggested that exercise training can reduce lung damage and improve dyspnea in mice with bleomycin-induced pulmonary fibrosis, thereby delaying the development of IPF [8]. The benefits of exercise training for IPF patients have been recognized, and more and more physicians are recommending it. In light of the fact that PR is an effective and safe tool for the management of IPF, an increasing number of physicians recommend that Baduanjin, a traditional Chinese exercise therapy, be utilized in the PR of IPF patients [9]. Nowadays, its indications are gradually expanding due to its functionality and practicability, which emphasizes not only the interaction between body posture and movement but also the harmony of meditation and breathing techniques. To engage in effective physical activity and mental concentration [10].

Preliminary experimental studies indicate that traditional Chinese exercise is a mild, self-healing, and functional exercise that is primarily characterized by isometric contraction, stretching, relaxation, and adjustment of body posture, combined with breathing techniques, with the primary goal of enhancing physical fitness. A meta-analysis revealed that traditional Chinese exercise is a low- to the moderate-intensity form of exercise that is appropriate for middle-aged and elderly patients with IPF and had a positive effect on lung function (improved FEV1 and FEV1/FVC as indicators), exercise capacity, and quality of life (6MWT) [11]. More and more studies attempt to explore this new application of traditional Chinese exercise in the prevention and control of chronic diseases [12]. pulmonary rehabilitation (PR) is an important component of non-pharmacological treatment for patients with IPF, Studies have shown that aerobic and breathing training appears to be complementary to exercise training, and pulmonary rehabilitation can prolong stressful exercise time in IPF patients, and improve dyspnea and health-related quality of life (HRQoL) in IPF patients [13]. However, some studies have demonstrated that routine tests are unnecessary for additional RCTs comparing pulmonary rehabilitation with standard care and that a new subgroup analysis of pulmonary rehabilitation is more appropriate.

Baduanjin is a kind of qigong exercise that originated in ancient China and integrates body and mind. Combining body and mind maximizes physical and mental health [14] and reduces anxiety, depression [15], physical pain, and other uncomfortable symptoms. Baduanjin emphasizes physical exercise and mental concentration, the connection between symmetrical body posture and movement, and breathing in a meditative and harmonious way. In addition, it has low physical and cognitive demands and is an aerobic exercise of moderate intensity that is suitable for home rehabilitation [16]. Based on its characteristics, it is very suitable for patients with pulmonary fibrosis and special groups such as weakened lung function [17] and has better compliance for the prevention and treatment of chronic diseases. Baduanjin exercise can improve exercise capacity, lung function, and quality of life in patients with chronic obstructive pulmonary disease, in part due to its appropriate intensity and pattern of intermittent intensity. Baduanjin could serve as an alternative to current exercise rehabilitation programs [18]. Therefore, it has been widely used in chronic respiratory diseases and other diseases.

Surprisingly few studies have focused on the critically required positions for recovery in IPF patients who, to avoid exertional dyspnea, frequently adopt a sedentary lifestyle, which predictably results in extensive skeletal muscle degeneration, social isolation, and its negative psychological sequelae [19]. The inhaled and exhaled gases of 28 healthy participants are collected for 45 min (15 min in each position) while they performed three different positions (lying, sitting, standing) in random order. It is determined that standing expends more energy than lying down or sitting, and patients with IPF are frequently forced to sit or lie down to avoid shortness of breath due to impaired lung function [20]. Patients with IPF are significantly less active than their age-matched healthy counterparts. As sedentary time increased, its lower daily physical activity led to a significant decline in survival [21]. The traditional Baduanjin has only one standing position, indicating that we must not only develop a pulmonary rehabilitation program that allows IPF patients to exercise while sitting or lying down in addition to standing, but also minimize prolonged sitting or lying down time in order to improve IPF patient survival. Liu et al. develop a simplified Baduanjin exercise scheme.

In this study, three distinct forms of Baduanjin, including sitting horizontally and vertically, are developed to accommodate IPF patients with varying disease severity; each form has its own distinctive characteristics. As an illustration, the seventh form of the vertical Baduanjin is a combination of the traditional Baduanjin and the “tiger rushing” movement in Wuqinxi. During the exercise, the hands are presented in the form of “tiger claws”, raised to the chest with a deep inhalation to make the sound of “si”, and the process of pushing the hands forward with force, accompanied by the exhalation of the gas, “hu” the sound of. Throughout the breathing process, when inhaling, the diaphragm and intercostal muscles contract, causing the anteroposterior, left and right, and superoinferior diameters of the thoracic cavity to increase, and the thoracic volume expands, causing the air pressure in the lungs to be lower than the outside atmospheric pressure, forming an active inhalation movement. When exhaling, the diaphragmatic muscles and external intercostal muscles relax, causing the thoracic volume to shrink and the lungs to retract, causing the air pressure in the lungs to be greater than the outside air pressure, resulting in passive exhalation. The combination of exhalation exercise and inhalation exercise maintains the continuity and patency of breathing. There is experimental evidence to show that when IPF patients are breathing, the activity rate of the diaphragm is lower than that of the healthy control group, especially when taking a deep breath. To achieve the effect of improving pulmonary ventilation [22]. This may fulfill the function of acute oxygen supplementation, which increases supplemental oxygen tolerance and alleviates dyspnea in individuals with idiopathic pulmonary fibrosis who do not have hypoxemia at rest [23]. At the same time, hypoxia may be related to the peripheral skeletal muscle dysfunction observed in IPF patients [24], so in the rehabilitation of IPF patients, hypoxia and skeletal muscle may have a mutual influence rather than an independent relationship. The fifth pose of Sitting Baduanjin is a combination of the traditional Baduanjin and Yijinjing movements of “drawing the knife”. Place one hand on the neck and the other on the waist and perform the exercise with comparable force using both hands. Through the external and internal rotation of the arms, similar to the action of expanding the chest, the intercostal muscles are contracted and the ribs are lifted, causing the anteroposterior, left and right, and superoinferior diameters of the thoracic cavity to expand and contract, delaying the reduction of chest-related muscles. Studies have confirmed that when the thoracic skeletal muscle mass of IPF patients reaches the level of low muscle mass, it is positively correlated with the quality of survival and prognosis of the patients. Therefore, the exercise of the thoracic skeletal muscle can improve the quality of life and the prognosis of patients [3]. The eighth form of horizontal Baduanjin is “bend the knee joint and dorsi-extend the foot joint”, which is to carry out related exercises for the patient’s limb function. During this session, engage the relevant limb muscles by flexing the knee and hooking the foot to prevent and improve muscle atrophy. Modern research has demonstrated that, with regard to skeletal muscle, appropriate exercise training can have an effect on peripheral skeletal muscle strength and limb mobility [25]. Simultaneously, the majority of anti-fibrotic IPF patients died of chronic respiratory failure; skeletal muscle loss is observed in these individuals, but there is no significant weight loss, which displays that preventing skeletal muscle atrophy in IPF patients is crucial [26].

In this study, five randomly assigned groups are combined with different interventions to evaluate their effects on lung function and motor function in IPF. Because the Modified Baduanjin is easy to learn, easy to operate, easy to carry out, simple, safe, and effective, it is necessary to further study the rehabilitation effect of the Modified Baduanjin on IPF patients. We expect the recommended exercise approach to considerably improve lung function, limb movement, quality of life, and psychological function, and to be applicable to the prevention, treatment, and rehabilitation activities of doctors.

## Methods

### Study design

This is a single-blind, randomized controlled clinical trial comparing (1) conventional care, (2) traditional Baduanjin (3) Modified Baduanjin (4) resistance training, (5) Modified Baduanjin, and resistance training. All the participants are from the Department of Respiratory Medicine, The First Affiliated Hospital of Jiangxi University of Traditional Chinese Medicine. On January 12, 2022, the protocol has been registered in the Chinese Clinical Trial Registry (ChiCTR2200055559). The study has been also approved on February 23, 2022, by the Ethics Committee of the First Affiliated Hospital of Jiangxi University of Traditional Chinese Medicine; the approval number is JZFYLL202202230011. All participants will sign informed consent forms in writing.

**Fig. 1:**
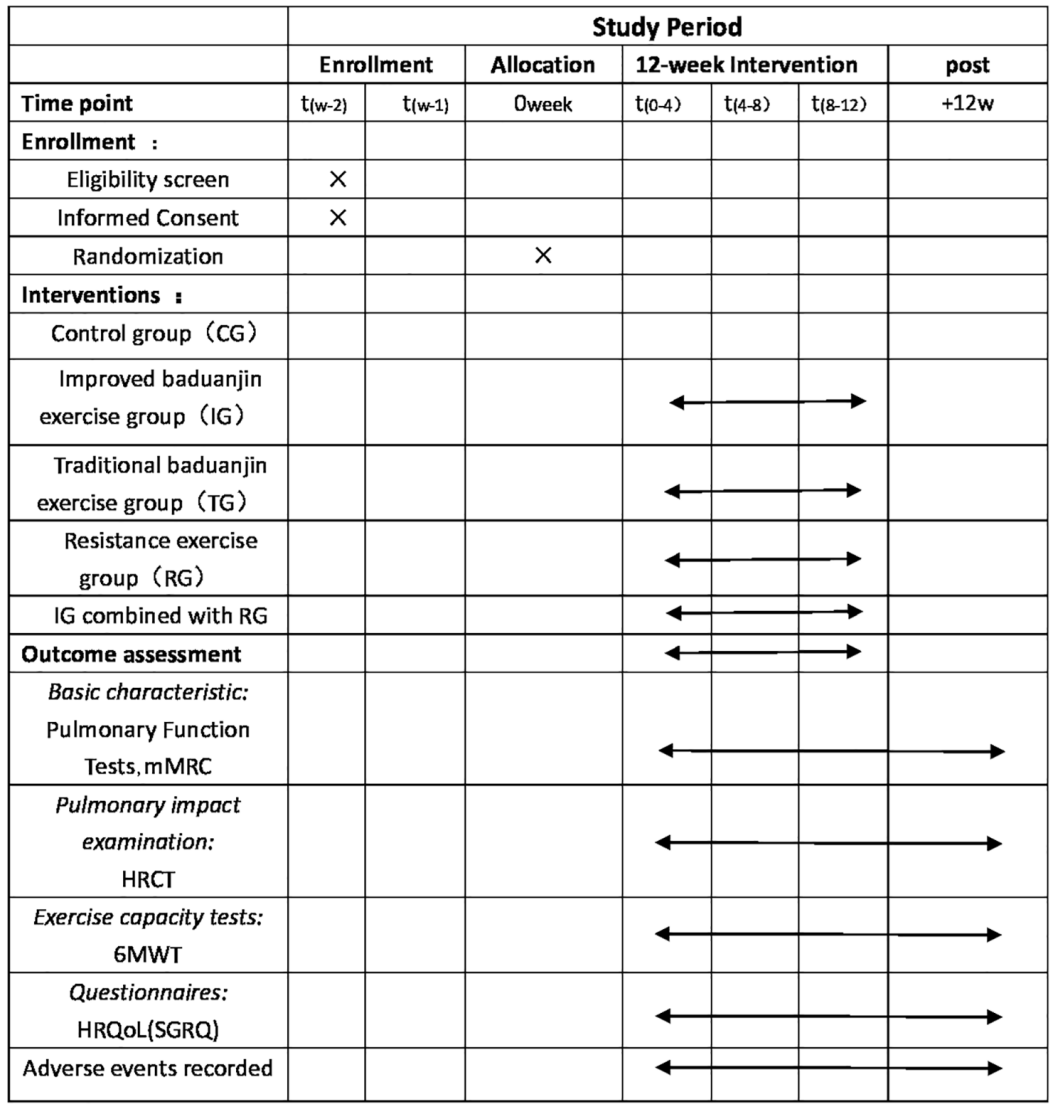
The study period

**Fig. 2:**
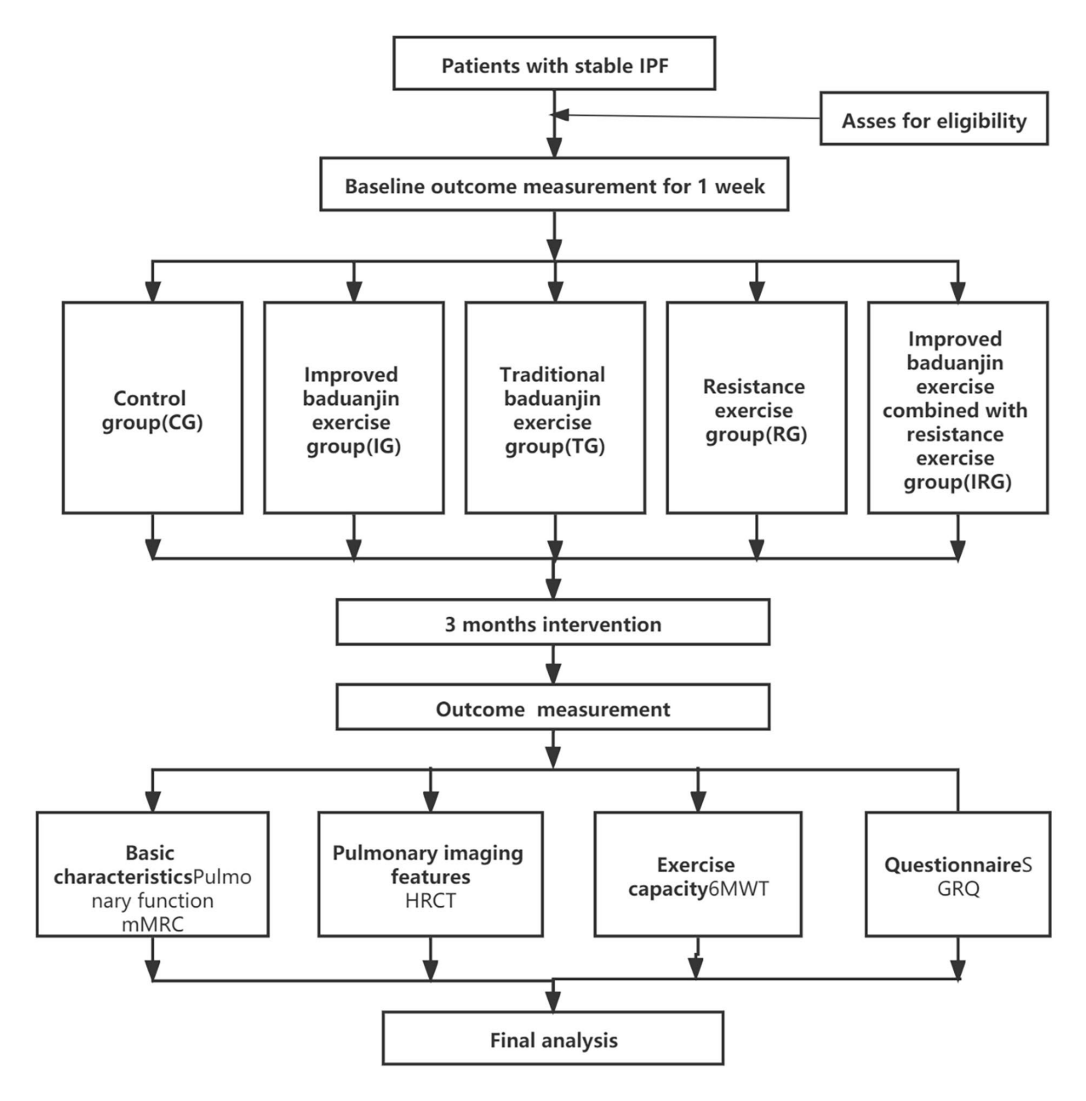
The study procedure

Subjects will be assigned according to a computer-generated table of random numbers and will be randomly assigned to 5 groups by an independent person who is unaware of the purpose of the experiment and the recruitment process. Sealed opaque envelopes are used to hide randomly assigned numbers to perform blinding. Considering that the intervention in this study has been performed in a motor manner, it is not possible to complete blinds for participants and therapists, so we included an outcome evaluator to keep the blinds going. The time points of the study are in Fig. 1 and the study procedure is shown in Fig. 2. The study protocol has been designed following the Standard Protocol Items: Recommendations for Interventional Trials (SPIRIT) guidelines [27].

### Study setting and recruitment

The experiment will be conducted in the Department of Pulmonary Diseases of the First Affiliated Hospital of Jiangxi University of Traditional Chinese Medicine. Relevant recruitment information will be announced through multiple social media and municipal advertisements. Through formal and informal invitations, participants’ names, ages, telephone numbers, and other relevant information will be registered to facilitate further screening. All willing to participate in the study of the participants will receive our leaflet, we will related information and the advantage of the participants of the study, the rights, interests, responsibilities, and obligations to print on the leaflets in order to effectively inform participants, we will be the first time, in the form of telephone invite all participants to the hospital to complete the test screening, The relevant baseline data and physical examination information of participants who met the inclusion criteria are recorded promptly. Participants will be recruited continuously until the required sample size is reached.

### Sample size

6MWT is the most commonly used outcome indicator in pulmonary rehabilitation (PR) and can be used for sample size estimation. In 6MWT, the minimum difference (miniDIFF) between different groups is 54 m, and the baseline standard deviation (SD) is 57 m. Given a two-tailed 0.05 and a power of 80%, the minimal sample size for each group is 19. To compensate for the expected loss of participants, an adjusted sample size of 27 could be acquired for each group, and 140 patients could be recruited, assuming a roll-out rate of 30%.

### Participants

All participants are stable IPF patients will be recruited from the Department of Respiratory Medicine at the First Affiliated Hospital of Jiangxi University of Traditional Chinese Medicine. Potential participants will be screened through the hospital’s clinical database to determine whether they will be contacted by phone or in-person for the study. All participants who signed the consent form will be evaluated by an assessor with their eyes covered. Participants who meet the relevant eligibility criteria will have their demographic, anthropometric, and questionnaire information recorded, and they will be randomly assigned to each group.

Diagnosis of IPF will be confirmed according to the Global IPF Initiative (GOLD) standard. The criteria include: (1) Age between 35 and 80; (2) The clinical symptoms are stable in the last two months; (3) Patients diagnosed with IPF who satisfied the diagnostic criteria of ATS/ERS/JRS/ALAT 2018 [According to past medical history, laboratory tests and HRCT (typical UIP and Probable UIP)]; (4) Have not participated in systematic or intermittent training for another sport in the past six months (no more than twice a week); (5) No other disabling diseases (e.g. stroke, Parkinson’s disease, etc.); (6) Patients who agree to sign written informed consent.

Among the exclusion criteria are the following: (1) other chronic respiratory diseases; (2) serious complications, such as cardiovascular, liver, or kidney disease; (3) in patients whose condition is rapidly deteriorating or urgently requiring other medication management; (4) skeletal muscle diseases or other diseases that may affect muscle strength assessment.

### Randomization and concealed allocation

Prior to the baseline assessment, participants will be randomized into five groups: CG, IG, TG, RG, and IRG. The allocation sequence will use “study randomized” transcendental prior distribution, the random number generator is (https://www.randomizer.org/) online random number generator, and the participation of participant recruitment assessment or not engage in the researchers. Concealed allocation will be achieved using sequentially numbered, sealed, and opaque envelopes kept in security cabinets that can only be opened by specifically authorized staff.

### Blinding (masking)

The outpatient doctors (S.W.K. and L.S.M) will conduct the initial assessment of the patients from the outpatient department, enter the collected information independently, and compare the information to find errors and correct them in time. The treating physicians (Z.H.W. and Z.N.W.) are blinded to the initial evaluations and group assignments. In addition to counting and analyzing the data independently of the multiple physicians administering the intervention, the statistical analysis team will be blind to the group assignments and the purpose of the study. Unmasking occurred upon completion of the trial or the occurrence of a serious adverse event.

### Intervention

**Fig. 3:**
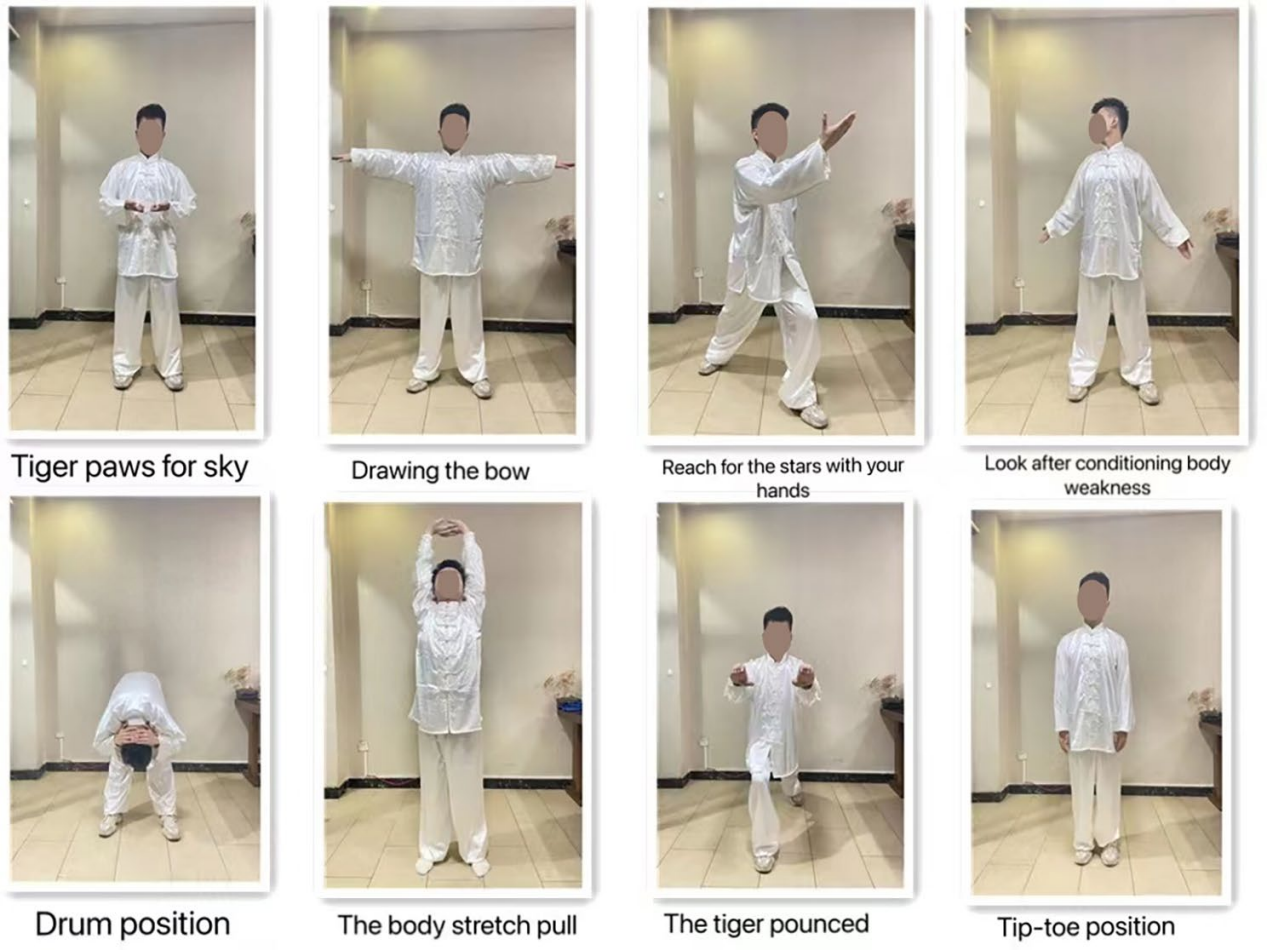
An improved version of the standing form Baduanjin consists of eight parts, which are: Tiger paws for sky, Drawing the bow, Reach for the stars with your hands, Look after conditioning body weakness, Drum position, The body stretch pull, The tiger pounced and Tip-toe position

**Fig. 4:**
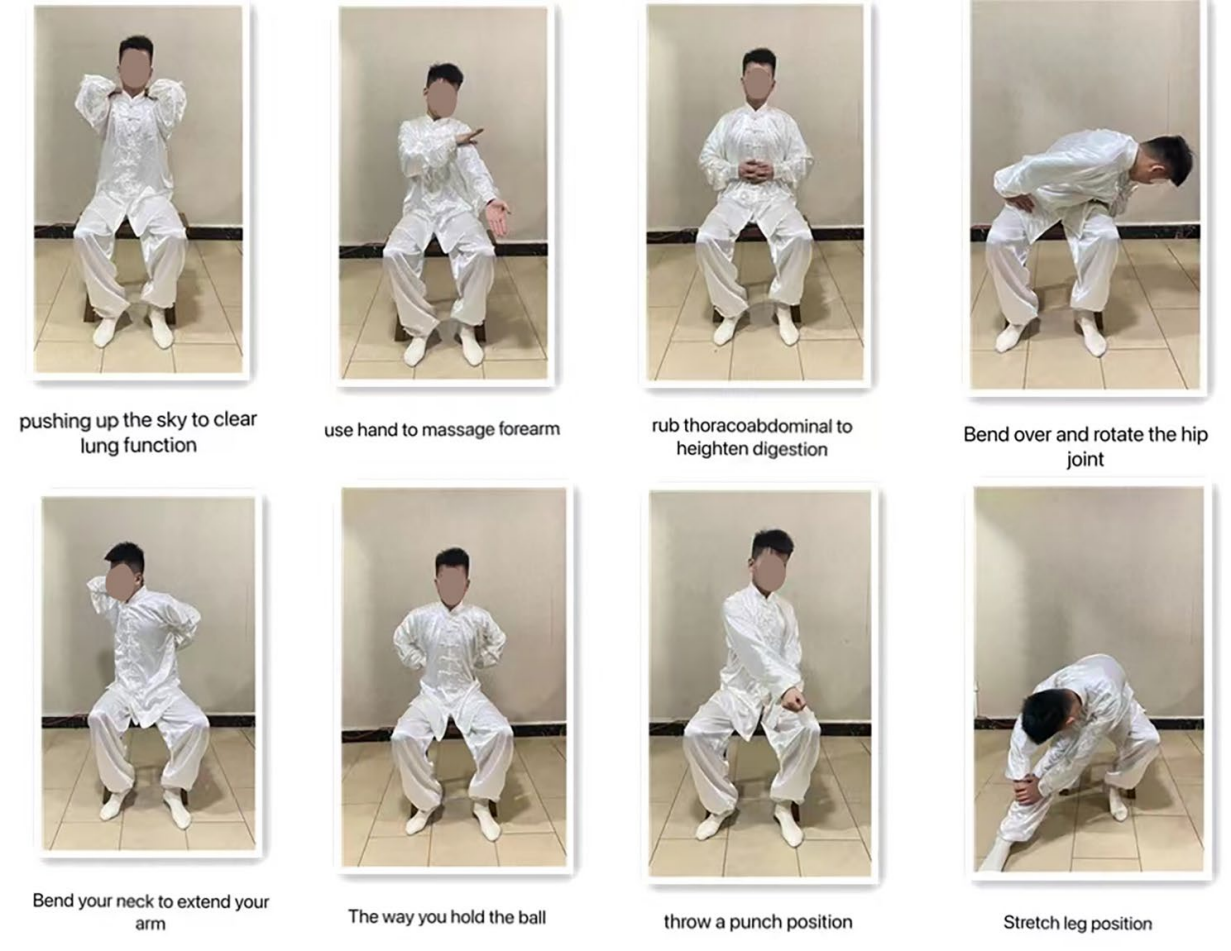
An improved version of the sitting form Baduanjin consists of eight parts, which are: Pushing up he sky to clear lung function, Use hand to massage forearm, Rub thoracoabdominal to heighten digestion, Bend over and rotate the hip joint, Bend your neck to extend your arm, The way you hold the ball, Throw a punch position and Stretch leg position

**Fig. 5:**
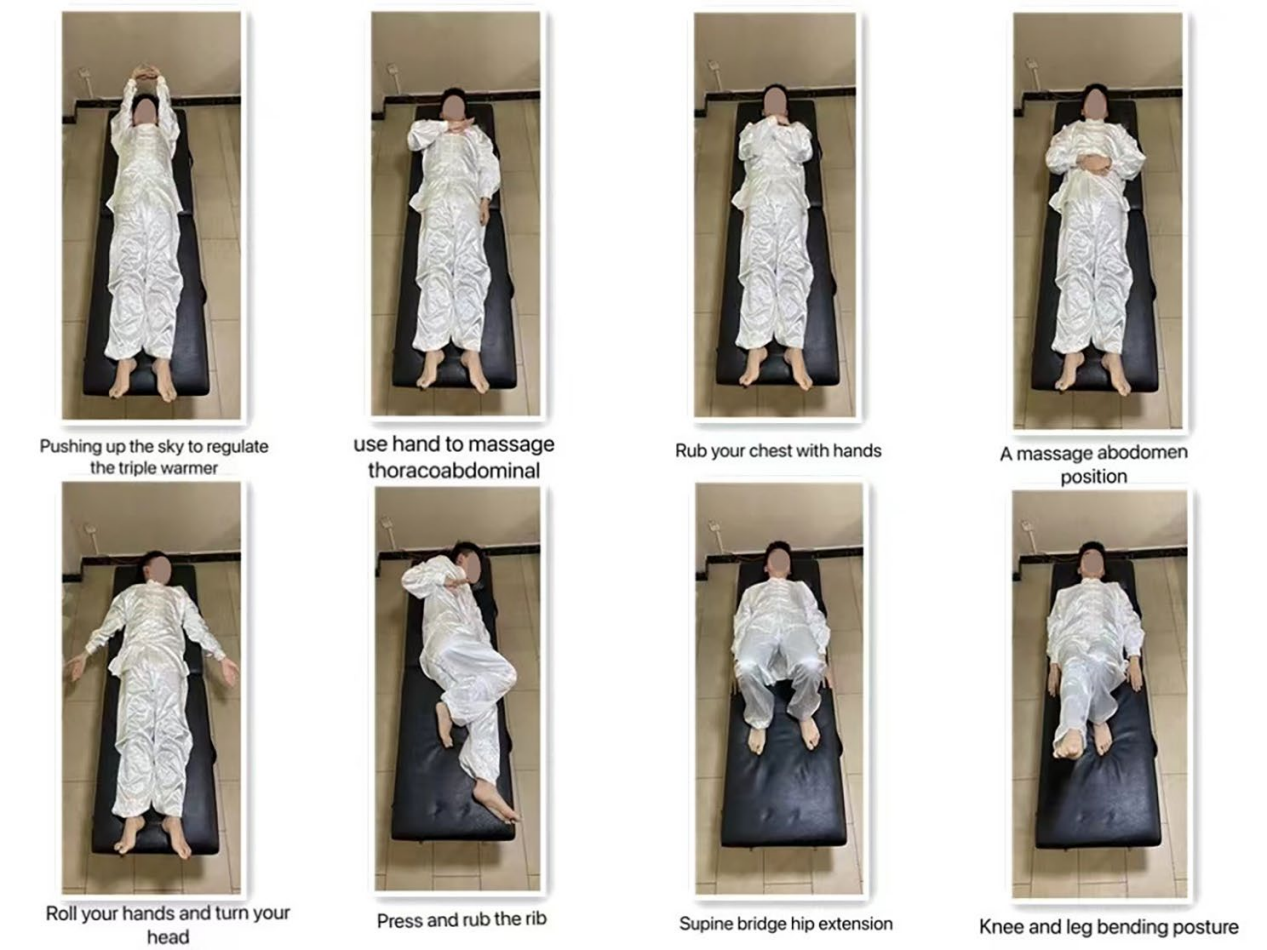
An improved version of the lying form Baduanjin consists of eight parts, which are: Pushing up the sky to regulate the triple warmer, Use hand to massage thoracoabdominal, Rub your chest with hands, A massage abdomen position, Roll your hands and tum your head, Press and rub the rib, Supine bridge hip extension and Knee leg bending posture

We will regularly provide verbal and visual instructions to all subjects, standardize the movement, strengthen the concept of movement, and establish requirements for the amount and frequency of movement. We will according to the specific needs of each group of the experiment accordingly the intervention wording, to distribute it to all team members to participate in, and all the subjects will be asked to record the action content, and exercise duration/sports strength/sports venues, as well as their movement during or after personal feelings, etc. The specific exercise movements of the three positions of the new Baduanjin are shown in these figures (Figs. 3, 4 and 5).

#### Control group

Members of the CG will continue to receive their regular care or medication without intervention for exercise.

#### Modified baduanjin exercise group

IG participants will continue to receive their existing prescription medication, plus an exercise program for three months. IG’s exercise regimen is set to be once a day and twice a day. A steering committee of hospital specialists facilitates weekly training and education courses. Each session will be 60 min in length. Include (1) 10 min of warm-up exercise; (2) 40-minute sports training of Modified Baduanjin; (3) 10 min of relaxing stretching and cooling exercises. Under the doctor’s verbal and visual supervision, the exercise is conducted safely, and their exercise intensity and heart rate are monitored to prevent accidents and ensure the trial ran smoothly.

#### Traditional baduanjin exercise group

In addition to the 3-month exercise program intervention, TG participants did not alter their original prescription medications. The frequency of TG’s exercise is once per day, twice per day. The hospital’s experts will form a steering committee to facilitate weekly training and education courses for TG participants. Each session will be 60 min in length. Include (1) 10 min of warm-up exercise; (2) 40 min of traditional Baduanjin exercise training; (3) 10 min of relaxing stretching and cooling exercises. Patient surveillance and patient safety are used in the same way as IG.

#### Resistance exercise group

In addition to resistance training, participants in the RG will continue to take their prescribed medications for three months. We will report on resistance training involving the trapezius/deltoid/biceps/pectoralis major, rectus femoris, gluteus maximus, quadriceps femoris, gastrocnemius, and soleus in previous studies of COPD patients with improved lung and limb function. The intensity of resistance training will be divided into eight levels, with the resistance increasing as the level increases. Three times per week, RG will exercise for one hour. Specifically include: (1) 10 min of warm-up exercise, (2) can involve the above 10 muscles-based resistance training, lasting 40 min. (3) 10 min of related muscle relaxation. Patient surveillance and patient safety are used in the same way as IG.

#### Modified baduanjin exercise combined with the resistance exercise group

Participants in the IRG will continue to receive prescription medications in addition to three months of Modified Baduanjin and anti-resistance exercises. The exercise program of IRG is set as Baduanjin for 7 days a week and anti-resistance exercise for 3 days a week, with the corresponding exercise twice a day. In addition, once a week, patients will engage in a prescribed training exercise under the supervision of doctors in order to standardize and supervise their exercise. Each session will last 60 min and include: (1) a 10-minute warm-up starter; (2) a 20-minute Modified Baduanjin exercise, the operation is the same as IG group; (3) rest for 5 min. (4) anti-resistance exercise for 20 min. The operation is the same as the RG group. (5) 5 min of relaxation and cooling exercise.

### Advice to stay active

Throughout the duration of the trial, both experimental and control participants will be required to attend an educational course on IPF treatment in order to maintain their interest and keep them active. The course will concentrate on two aspects: exercise therapy regimen for IPF and the positive effects of exercise on IPF. All participants are required to attend and sign in for the twice-monthly online and offline classes. The doctors responsible for teaching the course (M.L.Q and G.S.Z) will strongly encourage all participants to complete the course and meticulously record the completion of everyone’s course. The purpose of the course is to encourage participants to participate more actively in the experiment’s launch and completion of the corresponding indicator detection, which will facilitate the experiment’s execution and receipt collection.

### Adherence to interventions

To let all subjects complete the test, we will organize experts on the subjects not less than once a week phone access, answer the questions according to measured by the number of treatment adherence, and compliance of index is set in greater than or equal to 85%, the equivalent of CG participants to answer questions not less than 11. For patients in the other intervention groups, compliance is quantified by the number of hospital-organized supervised training sessions they attended and the number of daily exercise logs they completed. Again, this compliance is set to at least 85% completion of the course or log.

### Outcome measurement

Outcomes will be measured at two-time points (within 1 week and after 3 months) for participants who complete baseline enrollment. Primary prognostic indicators included Lung function and 6MWT. Secondary outcome measures included mMRC, HRCT, and the questionnaire.

#### Assessments of basic characteristics

The patient will undergo lung function tests under the supervision of medical staff. TLC, VC, DLco, and min•mmHG are recorded in detail after the test [28, 29], while the lung function assessment table contains three levels (effective, stable, and ineffective) for qualitative assessment according to different values of TLC, VC, DLco, and min •mmHG.

The evaluation is based on the dyspnea scale (mMRC) developed by the British Medical Research Council [30]. The medical staff instructed the patients to complete the questionnaire according to protocol, and the patients are evaluated at the conclusion of the experiment. Each subject’s dyspnea level is meticulously recorded and categorized into three distinct categories. The severity of dyspnea increases as the dyspnea score rises.

#### HRCT of the chest

According to the 2018 New Evidence-based Guidelines for Diagnosis and Treatment of IPF [31], High-Resolution CT (HRCT) is the most important way to diagnose IPF at present [32]. The HRCT of the subjects before and after the trial is evaluated according to the “HRCT Efficacy Evaluation Table” and “HRCT Clinical Symptoms and Signs Efficacy Evaluation Table”. The evaluation criterion stated that the milder the lung lesions, the higher the HRCT score.

#### Exercise capacity tests

6MWT is mainly used to evaluate the severity and prognosis of heart and lung patients. The medical staff will introduce the test procedure to the patient and tell the patient to carry out the test after the patient calms down. Within 6 min, the patient began walking back and forth from the starting point. 15 s before the end of six minutes, the medical staff will inform the patient: “The test is coming to an end, please stand still when you hear stop.“ Borg grading is used after the test. 6WMT is divided into four grades according to the length of walking distance (300 m, 375 m, and 450 m as nodes). The longer the walking distance, the higher the grade. The Borg index (1–10) measures dyspnea or fatigue after a 6-minute walking test.

#### Questionnaires

The health-related quality of life (HRQoL) uses the St. George Respiratory Questionnaire (SGRQ) [33]. The SCRQ consists of 50 questions that are independently completed by the patient on the day of the Lung function examination, with no reminders and an observer checking for omissions. Each question has a predetermined score, and the weighted average calculation method will be utilized. The greater the score, the more severe the life consequences. Each part’s score is calculated, and the final score is obtained following processing.

### Criteria for discontinuing or modifying allocated interventions

We will seek consent from participants and conduct the trial after signing the informed consent form, and the individual wishes of the participants will be considered. If the participants do not consent and refuse to participate in the experiment, we will respect their views, postpone or alter the intervention, and report the exact reasons. The trial will be stopped if a participant is exposed to harmful conditions in the event of a possible or impossible adverse event or emergency (COVID-19 infection, acute exacerbation of illness, life-threatening situation). We will take proactive steps to provide appropriate treatment and care to patients who have adverse events during the trial.

### Statistical analysis

The SPSS (version 23.0) is used for data management and statistical analysis. The kolmogorov-Smirnov test is used for normality test. The normality data are expressed as mean ± SD, and the skewness data are expressed as median and quartile spacing. An intention-to-treat analysis will be used to assign participants whether or not they complete the intervention. Lost result data will be processed using a mixed-mode approach. Each protocol is then examined, including only patients who completed all outcome measures and followed the intervention protocol. Comparisons of baseline differences between groups will be made using one-way analysis of variance (ANOVA) or nonparametric tests. The training-related effects are evaluated using a 2 × 2 ANOVA (group × time). Differences in each group of variables over time (time effect, from baseline to 3 months) will be assessed using either a paired T-test or a nonparametric test. To detect differences between categorical variables, the Chi-square test will be applied. Bilateral *P* values < 0.05 are considered significant. In addition, the effects of different interventions on Lung function have been found in previous studies, and disease severity may be an important factor. Therefore, in order to investigate differences in effectiveness based on disease severity, a pre-designated subgroup analysis will be conducted.

#### Data analysis

The SPSS (version 23.0) is used for data management and statistical analysis. The kolmogorov-Smirnov test is used for normality test. The normality data are expressed as mean ± standard deviation, and the skewness data are expressed as median and quartile spacing. An intention-to-treat analysis will be used to assign participants whether or not they complete the intervention. Lost result data will be processed using a mixed-mode approach. Each protocol is then analyzed, including only subjects who completed all outcome measures and followed the intervention protocol. One-way analysis of variance (ANOVA) or nonparametric tests wil be used to compare baseline differences between groups. The effects of training are evaluated using a 22 ANOVA (group × time). Using either a paired T-test or a nonparametric test, differences in each group of variables over time (from baseline to three months) will be evaluated. To detect differences between categorical variables, the Chi-square test will be applied. P values less than 0.05 are considered significant.

#### Subgroup analyses

Based on a previous statistical analysis of the results of pulmonary function tests, disease severity may be a potential factor contributing to the differences in outcome measures. Therefore, we considered performing corresponding subgroup analyses. Further subgroup analyses by sitting, standing, and lying will be considered to determine whether the effect of this exercise therapy on the treatment of IPF varies by posture.

#### Sensitivity analysis

Sensitivity analyses are performed to assess the robustness of the primary analysis using multiple imputations and assumptions regarding the missing data.

### Safety

All the exercise is completed under the supervision of the designated doctor, and the patient’s heart rate is monitored synchronously. When the patient’s heart rate reached 80% of their reserve heart rate, the patient would exercise temporarily, the heart rate monitoring would be maintained, and the patient could continue to participate in the test until the stability. Patients who feel various kinds of discomfort can request a temporary test and receive the timeliest corresponding treatment. In the event of an emergency, including but not limited to chest pain, extreme difficulty breathing, nausea, dizziness, or injuries such as falls during exercise, patients will be asked to discontinue the trial. The experimenter will document the patient’s condition and make a clear determination regarding the trial’s continuation.

### Data collection and management

Baseline data will be collected, and baseline assessments will be completed prior to randomization. All data and results are collected on paper in a uniform format and converted to electronic documentation by dedicated research assistants. The same data will be re-entered by a second research assistant, the completeness of the data will be verified by comparing it to the data entered by the first research assistant, and errors will be discovered and corrected on time. In the event of a challenging circumstance, the data expert and the project manager will discuss and resolve it.

To facilitate the continuous management of data and the confidentiality of the data, the individual participants will only be identified by the trial identification number. The data collected at all stages will be converted into electronic data and stored on a password-protected server to ensure data confidentiality, while the corresponding paper documents will be stored in a locked filing cabinet. Only authorized researchers are permitted to access the data. Each participant’s identity will be concealed and identified with a unique number to prevent the statistician from discovering personal information and group assignment. The result will be a group of data that prevents any personal data from being displayed to ensure confidentiality. The examiner will supervise the measurement of the completion and data of all questionnaires and clarify the uncertainty of the situation, which will take 30 min; the questionnaire and data quality will be guaranteed.

## Discussion

Dyspnea is a typical feature of IPF. In clinical practice, the majority of IPF patients experience varying degrees of limb function degradation as the disease progresses. Therefore, it is of utmost clinical importance to treat dyspnea and improve or enhance the limb function of IPF patients.

Baduanjin is a gentle, safe, and effective traditional Chinese sport. Compared with the traditional Baduanjin, the Modified Baduanjin has the following important characteristics: (1) Easy to operate, simple and interesting action, at the same time, we combined the features of the Modified Baduanjin to remember the name. (2) Easy to develop, the Modified Baduanjin has extremely low needs for the environment and site and does not borrow any tools. It can be carried out effectively at home, in the office, and even in a hospital bed. (3) Suitable for prevention, treatment, and rehabilitation of IPF, can be used in the complete process of IPF disease, and according to the severity of the disease, can choose different kinds of vertical, seated, and horizontal training. It is convenient for doctors to carry out their duties. Doctors can use the Modified Baduanjin in the propaganda, education, and prevention and treatment of IPF diseases. Patient training at the same time can promote communication between patients and patients, patients and doctors, compared with drug therapy, no pain, this is a natural, pollution-free, easy, and pressure-free new way of prevention and treatment. This study, therefore, focuses on the lung function and limb motor function of stable IPF patients and measures the effect and effect of Modified Baduanjin on IPF patients using different indicators.

Rehabilitation exercise is an important part of IPF treatment. Anti-resistance exercise stimulates and trains the muscles related to IPF rehabilitation through a variety of tools, such as instruments, weight lifting, rubber bands, and so on, to improve the skeletal muscle strength and function of IPF patients. COPD patients who use rubber band exercises can improve their 6MWT test results, according to studies. We hope to examine the effect of anti-resistance exercise on IPF patients and compare its therapeutic effect to that of Modified Baduanjin in order to determine its specific effect and effect on stable IPF.

Resistance movement is characterized by setting relevant resistance movements for the relevant ten muscles, to improve the muscle strength and function of the relevant muscles, and to improve lung function and limb movement function. The Modified Baduanjin is a combination of whole-body aerobic exercise and breathing that can effectively improve dyspnea and limb movement ability. Consequently, we hypothesize that this combined exercise regimen may have a synergistic effect on IPF patients.

This study has several bright spots. First, the Modified Baduanjin is innovatively developed in three forms, standing, sitting, and horizontal, compared with traditional Chinese sports skills. As far as we know, the latter two forms are new forms that are not available in other exercise therapies. Therefore, patients with severe lung function injury, particularly long-term hospitalized patients, can choose the appropriate sitting or horizontal rehabilitation posture, which is not possible with other exercise therapies. Second, for IPF patients, we evaluate several comprehensive indicators of new-type Baduanjin on IPF in the stable phase, paying special attention to lung function and limb function. Third, a subgroup analysis of patients with different degrees of disease is conducted to study whether there are differences in the therapeutic effects of vertical, sitting, and horizontal types respectively, and finally to find out the best combination plan to optimize the therapeutic effect. Finally, this study is conducted strictly and standardized under CONSORT guidelines [34], to explore the therapeutic effect of Modified Baduanjin on stable IPF patients.

Still, our study has some limitations. First of all, as a kind of exercise intervention, the Modified Baduanjin will inevitably fail to blind participants and doctors, which may lead to a certain degree of bias in the results. Lack of blinding may also contribute to sample shedding, with participants in different treatment groups experiencing varying levels of psychological impact, leading to the possibility of early withdrawal from the trial. Second, the focus of this study is given priority to home exercise intervention, the patient’s compliance may not be able to get a good guarantee, but the staff will promptly with the participants of this study via remote video and telephone contact, strengthen communication, supervision, and education curriculum implementation once a week, at the same time to test the normal; Lastly, the promotion of the Modified Baduanjin may be limited to some extent by the specificity of different populations as a result of regionalism, as different regions and populations have varying degrees of acceptance and recognition of the Modified Baduanjin.

In conclusion, this study introduced a Modified Baduanjin through three different forms (vertical, seated, horizontal) on the stability of IPF patients’ intervention of clinical trial plan, aims to investigate the lung function and limb function and role in IPF patients, as well as the influence of a single solution (including Modified Baduanjin and resistance training) and the benefits and drawbacks of the joint scheme. In addition, these results may provide clinicians and therapists with a new IPF rehabilitation protocol that is simple, safe, and effective.

### Plans for communicating important protocol amendments to relevant parties

Changes to the protocol, such as inclusion and exclusion criteria, efficacy evaluation, and outcome indicators, should be agreed upon and approved by the relevant parties (ethics committee, trial participants), and the corresponding notification obligation should be carried out. The changes related to participants should be notified place, and their feedback information should be collected. At the same time, the satisfaction of participants should be collected through questionnaires.

### Trial status

At the time of submission, recruitment for the trial has been completed. The first participant was included on 4 April 2022.

## Data Availability

Not applicable.

## Abbreviations

6MWT: 6-min walking test
ATS/ERS/JRS/ALAT: American Thoracic Society/European Respiratory Society/Japanese Respiratory Society/Latin American Thoracic Society
CG: Control group
DLco: Diffusion capacity for carbon monoxide of the lung
FEV1: Forced expiratory volume in 1 s
FVC: Forced vital capacity
GOLD: Global Initiative for Chronic Obstructive Lung Disease
HRCT: High-resolution CT
IG: Modified Baduanjin exercise group
IPF: Idiopathic Pulmonary Fibrosis disease
IRG: Modified Baduanjin exercise combined with the resistance exercise group.
mMRC: Modified Medical Research Council Dyspnea Scale
PR: Pulmonary rehabilitation
TLC: Total Lung Capacity
VC: Vital Capacity
